# Self-Regulation and Wellbeing When Facing a Blocked Parenthood Goal: A Systematic Review and Meta-Analysis

**DOI:** 10.1371/journal.pone.0157649

**Published:** 2016-06-23

**Authors:** Sara Mesquita da Silva, Jacky Boivin, Sofia Gameiro

**Affiliations:** School of Psychology, Cardiff University, Cardiff, United Kingdom; Harbin Medical University, CHINA

## Abstract

Developmental regulation theories claim that continuing to pursue a goal when it becomes blocked contributes to poorer wellbeing. This consequence is expected to lead to the use of self-regulation strategies in the form of higher disengagement from the goal and higher reengagement in other meaningful goals. The use of these strategies is expected to lead to better wellbeing. A systematic-review and meta-analyses were conducted to test the major predictions of developmental regulation theories for blocked parenthood goal and to investigate possible moderator variables, particularly type and degree of blockage. A total of eight meta-analyses were performed using random-effects models. Moderation was tested with subgroup analysis. After searching eight databases, 4977 potential relevant manuscripts were identified but only six met inclusion criteria. From the eight meta-analyses conducted, only two were significant. In line with prediction, higher goal blockage was related to higher negative mood and reengagement in other life goals was associated to higher positive mood (*p* < .001). From a total of eight subgroup analyses performed, results showed that disengaging had a positive impact on wellbeing for people experiencing an unanticipated type of blockage (i.e., infertility) but not for those with an anticipated one (i.e., postponing parenthood; *X2* = 4.867, *p* = .03). From the total of twelve sensitivity analyses performed only one suggested that results might differ. The association between disengagement and mood varied according to study quality. When only average studies were included this association was negative, although non-significant. The evidence obtained did not fully support developmental regulation theories for the pursuit of parenthood goal, but primary research had too many methodological limitations to reach firm conclusions. Future studies aimed at investigating blocked parenthood goal are required to evaluate the value of developmental regulation theories.

## Introduction

Life is shaped around defining and pursing important goals. The achievement of these goals gives meaning to people’s lives. Individuals develop self-regulation strategies to increase their chances of achieving life goals [1]. Self-regulation strategies emerge from a dynamic motivational system that allows people to prioritize their goals and also to match goals to opportunities to achieve a better fit with their environment [2–4]. An adaptive self-regulation strategy should maximise goal achievement and lead to positive states of wellbeing [1,5,6].

In the last two decades of research, the conceptualization of adaptive self-regulation has been shaped according to three major emerging developmental regulation theories [4]: the *Dual-Process Model of Assimilative and Accommodative Coping* [7], the *Model of Selection*, *Optimization and Compensation* (SOC model) [5], and the *Motivational Theory of Life-Span Development* (MTD) [8]. These are complex theories that explain how people adaptively regulate goal pursuit during their life-span. Initially, research on self-regulation assumed that adaptive regulation depended on individuals’ persistence in pursuing important life goals [9,10]. More recent developmental regulation theories (such as the *Dual-Process Model of Assimilative and Accommodative Coping*, the SOC model, and the MTD) have introduced the idea that adaptive self-regulation also depends on timely disengagement, which is the capacity to withdraw engagement from goals that cannot be achieved (i.e., blocked goals). Numerous empirical studies [11–13] have been converging on a main finding that people employ two main self-regulation strategies in such situations: goal engagement and goal disengagement. The former corresponds to the ability to stay committed and invest further effort (e.g., time and energy) towards the initial goal individuals are pursuing, whereas the latter is related to the ability to withdraw commitment and effort (e.g., time and energy) from the initial goal when it becomes blocked. Therefore, these self-regulatory processes involve a cognitive process of commitment and a behavioural process of effort [6]. In addition, Wrosch et al. [6] proposed that individuals use a third self-regulation strategy, reengagement, which is the ability to invest in new alternative and meaningful life goals. A blocked goal, as developmental regulation theories propose, is likely to create demands on the individual. When these demands exceed the coping resources available (e.g., social support, finance, resilience) individuals will experience psychological stress [14] and may use coping strategies to address that stress. These strategies can facilitate self-regulation. For instance, individuals can just re-interpret a goal blockage to decrease its emotional impact (i.e., a type of antecedent-focused strategy named reappraisal [15]) and therefore decrease the need to engage with the goal. Despite the interplay between coping and self-regulation, coping theory is not the remit of this review. Nonetheless, future research should focus on better understanding how the use of different coping strategies may facilitate or hinder adaptive self-regulation.

Overall, developmental regulation theories seem to agree on three main theoretical predictions [4] that have already been tested in relation to different blocked goal situations, such as couple’s separation in late midlife [13], AIDS [16] and transition from school to work in adolescents [17]. The three predictions are that when people experience a blocked goal they: (1) experience poorer wellbeing; (2) start disengaging from the goal and reengaging with alternative life goals, and; (3) experience better wellbeing from using goal disengagement and goal reengagement self-regulation strategies. This dynamic cycle of adjustment-regulation-adjustment is expected to enable people to carry on with development. Concerning prediction one, previous research concluded that women who maintained a childwish after finishing fertility treatment presented worse adjustment than those who did not maintain it, independently of their parenthood status [18]. In relation to prediction two, research showed that adults suffering from long-term spinal cord injuries started disengaging from goals that were hard to achieve due to their health condition, such as becoming parents [19]. Finally, in respect to prediction three, a study showed that parents of children with cancer had lower levels of depression when they disengaged from the challenging situation they were facing or/and reengaged in alternative life goals, e.g., by redirecting their energy to other goals such as leisure activities [6].

Parenthood is a central life goal that 77% of people intend to achieve at some point in their lives [20]. However, many people are confronted with obstacles when trying to achieve parenthood. There are many kinds of blocked goals and developmental regulation theories do not address whether the type or degree of a blocked goal moderates the three predictions described. Most goals are age constrained, meaning that there are age periods in development when opportunities to achieve the goals are maximised, beyond which attainability starts to decrease until it is close to null [8]. Because developmental deadlines are known, this type of blocked goal can be anticipated and people can anticipate the need for building self-regulatory strategies. Anticipated blocked goals correspond to situations where people passed the age deadline when opportunities were optimal without taking action toward the desired goal. In contrast, unanticipated blocked goals result from unexpected negative life events, such as disease or disability, which disrupt the normative developmental process. In the case of [biological] parenthood, chances are maximised for women in their 20s but start to decrease rapidly beyond the age of 31 to become close to null after the 40s [21,22]. In Europe, women are also expected to have children around 24–25 years of age and in the worst case between 40 and 45 years of age [23]. These biological and social norms move people toward attaining their parenthood goal in their mid to late twenties, which corresponds to the current national mean age of 26.3 years in the US [24]. Some people can be faced with anticipated reasons for a blockage because they delay parenthood to prioritize other life goals such as career pursuits or a contemporary life-style with several alternatives to parenthood [25]. However, others can be faced with unanticipated reasons such as unexpected reproductive problems, more specifically infertility [26]. Infertility is defined as one year of regular unprotected sexual intercourse without a conception [27]. Main reasons for infertility are ovulatory disturbances caused by endocrinology abnormalities, diminished ovarian reserve [28] and poor semen quality [29]. It can be expected that individuals will find it harder to self-regulate in these cases than when blockage progressively develops with some form of awareness.

Another issue concerns the degree of blockage that people can experience. Developmental regulation theories propose that adaptive self-regulation is dependent on a person’s capacity to engage when there are still more opportunities than constraints to achieve a goal (i.e., when goal blockage is low) and to disengage and reengage in alternative life goals when there are fewer opportunities than constraints, i.e. when goal blockage is high [4,6]. These variations in the degree of goal blockage could be expected to influence the impact of goal blockage on wellbeing, the use of self-regulation strategies following a blocked goal and the effect of these strategies on subsequent wellbeing. If applied to the case of anticipated blockage to parenthood, developmental regulation theories would propose that increasing efforts to conceive while younger than 40 would be adaptive, but thereafter the most adaptive strategy would be to disengage from the pursuit of parenthood and reengage in other life goals that can still be achieved. In the case of unanticipated blockages such as infertility, developmental regulation theories propose that it would be adaptive to engage in fertility treatment while prognosis is good but thereafter individuals would better adjust by stopping treatment and engaging with other life goals. Success rates vary according to the number of fertility cycles and, presumably, so would perceived degree of goal blockage. After a typical course of 3 cycles, the chances to achieve pregnancy decrease [30]. Because in most western countries it takes a median of 2 years to complete 5 cycles [31], the degree of blockage could be conceptualized as low and high depending on whether patients were undergoing treatment for less or more than 1 year, respectively.

### The present study

The present meta-analyses aim at testing the three predictions of developmental regulation theories in the context of a blocked parenthood goal. More specifically, it is expected that (1) individuals facing a blocked parenthood goal will experience poorer wellbeing; (2) will disengage from parenthood goal and/or reengage with other meaningful goals and, finally, (3) will experience better wellbeing from using goal disengagement and goal reengagement strategies. Additionally, as a secondary aim, the present study also explored whether the associations hypothesized were moderated by type (unanticipated, anticipated) and degree (low, high) of goal blockage and study design (cross-sectional and quasi-experimental, longitudinal) and study quality (low, average, high).

## Methods

### Search Strategy

The present study adheres to the PRISMA (Preferred Reporting Items for Systematic Reviews and Meta-Analyses) guidelines for the reporting of systematic reviews and meta-analysis [32]. A review protocol does not exist.

The search terms were associated with goal blockage (e.g. “blocked” or “unattainable”) AND parenthood (e.g. “maternity”, “birth”) AND wellbeing (e.g. “quality of life”, “mental-health”) OR goal blockage (e.g. “blocked” or “unattainable”) AND parenthood (e.g. “maternity”, “birth”) AND developmental regulation strategies (e.g. “goal engagement”, “primary control”) (see table in S1 Table). Eight databases were searched: AMED (Allied and Complementary Medicine), EMBASE, HMIC (Health Management Information Consortium), ICONDA, Ovid MEDLINE(R), Ovid MEDLINE(R) In-Process & Other Non-Indexed Citations, PsycINFO and PsycArticles Full Text (1806 to February 2015). The literature search was limited to humans but had no language or publication type restrictions (journal, conference, paper or dissertation).

The processes of building the search strategy, study selection and duplicate exclusion were done by S.M.S. and S.G. and all papers then sifted for relevance based on their title and abstract. When these seemed relevant the full paper was accessed. Disagreements about inclusion were solved by discussion until consensus was reached. Reasons for exclusion were noted for all full papers accessed (see table in S2 Table). The reference lists of included studies were checked to identify additional relevant papers for inclusion. S.M.S. emailed the first authors to obtain full texts that could not be accessed via other methods (two book chapters, a PhD dissertation, and two journal articles).

### Eligibility Criteria

Four inclusion criteria were used. Studies were included if they were: (a) based on developmental regulation theories; (b) referred to the specific situation of parenthood goal blockage; (c) reported on at least one quantitative association (significant or not) among goal blockage, wellbeing and self-regulation strategies; and (d) reported original quantitative data. The use of developmental regulation theories was inferred if the authors used a valid (previously tested) theory as the rationale of the article.

### Data Extraction

The present data extraction process was developed in accordance with the Cochrane Handbook for Systematic Review of Interventions guidelines [33]. Data extraction was independently done by S.M.S and S.G. First, data related to the main study characteristics were extracted: country; sample (sample size, gender, mean age (*SD*), age range); sample characteristics (population and context); general aim and study design.

Data about the conceptualization and operationalization of the study variables were also extracted. This extraction included the theoretical framework (the use of a valid developmental regulation theory), the theoretical propositions tested (association between blocked parenthood goal and wellbeing, blocked goal and self-regulation strategies and self-regulation strategies and wellbeing) and the moderator variables (type and degree of goal blockage). The first moderator variable of the study was the type of goal blockage and it was categorized according to whether it was unanticipated or anticipated. Blocked parenthood goal was defined as being unanticipated for those participants who were facing an unexpected medical threat, i.e. when the sample referred to patients diagnosed with infertility, undergoing fertility treatment or who had already finished the treatment process. An anticipated blockage was considered when blockage was associated with the participants’ age because these participants were not facing an unexpected medical event and were probably dealing with the consequence of postponing parenthood. The second moderator variable was the degree of blockage participants were experiencing. For those assigned to an unanticipated blockage (i.e., infertility), the degree of blockage was considered low if the study sample was in the diagnostic phase, was undergoing a type of initial treatment such as IUI (*Intrauterine insemination*) or were undergoing IVF (*In-vitro fertilization*) or ICSI (*Intra-cytoplasmic sperm injection*) treatment for less than 12 months. Otherwise unanticipated blockage was considered high because a year is usually required to complete the typically recommended regimen of three *IVF* or *ICSI* cycles [31]. For those participants assigned to an anticipated blocked goal (i.e., age-related), the degree of blockage was defined as low when women were between 29–40 years of age and as high if women were over 40 years of age because fertility gets close to null after the age 40 years [21].

Third, data about the measures used in each study to assess goal blockage, wellbeing and self-regulation and their reliability were also extracted. These data included extracting whether the study used an objective (age and infertility diagnosis) or subjective measure of goal blockage (i.e., self-report items, e.g. “How blocked do you feel in your goal of becoming a parent?”) and which self-regulation strategies (goal disengagement and reengagement) were assessed. When a study operationalized self-regulation using multiple indicators of each self-regulation strategy (e.g., study 1 of Heckhausen et al. [34]), only one indicator for each strategy was extracted to perform the meta-analyses. The indicators were chosen according to the degree to which their content was relevant for each one of the self-regulation strategies included. Disagreements about content relevance of the indicators were solved by discussion. Data about the wellbeing measure were extracted and coded positive when it used positive affect, positive states of mind and life satisfaction and negative when it used depressive symptoms and negative affect. One of the first authors of the studies included was contacted in order to obtain data for the variables goal blockage, goal disengagement and goal reengagement and wellbeing that were not presented in the paper. Finally, to calculate pooled estimates of the associations tested, the study sample size, correlation coefficient(s) or standardized beta coefficient(s) for the test of association among self-regulation strategies, wellbeing and goal blockage was extracted from each paper.

### Quality Assessment

S.M.S. and S.G. assessed the quality of the included studies according to a quality assessment scale they developed. Four general quality criteria were used. Points for representativeness of the study sample (four points) were attributed if (a) the blockage to parenthood experienced by participants was clearly defined by the authors (one point); (b) more than 80% of eligible participants agreed to participate in the study (one point); (c) more than 80% of those who participated on the follow-up study completed the study (one point); and (d) correlates or predictors (main socio-demographic variables, such as age, gender, educational level and number of children) of study dropout were taken into account (one point). The adequate use of a theoretical framework was awarded a point if the aims and the hypotheses of the study were clearly derived from developmental regulation theories (one point). Measurement quality was considered to be valid if evidence of construct validity was provided, i.e. validated measures were used or data on construct validity in the study sample was provided (one point). Measures were considered reliable if the authors presented satisfactory values of internal consistency (*α* ≥ .70) for the study sample, or inter-rater reliability checks when using behavioural measures (one point). Finally, the evaluation of the study hypotheses was assessed with four criteria (four points): One point was awarded to studies that assessed causal relationships between variables by using a longitudinal or experimental design. Studies with enough power to detect significant statistical associations (i.e., small effect sizes) were also awarded one point. Power was determined by G*Power software [35]. Finally, we evaluated if all potential bias or unmeasured confounders were assessed (one point) and if the analytical plan allowed for the correct testing of the specified hypothesis (one point).

The overall quality rating was the sum of all met criteria and could range from zero to 11 points. The quality rating was divided in three main quality labels, low (scores from zero to four points), average (scores from five to nine points) and high (scores from 10 to 11 points).

### Data Analysis

Eight meta-analyses were performed to evaluate the direction and magnitude of the associations among goal blockage, negative mood, positive mood, goal disengagement and reengagement. Pooled effect size estimates of the associations examined were calculated using sample size, the correlation coefficient(s) or standardized beta coefficient(s). A random-effects model was chosen because it was assumed that each population of the studies included would reflect a different effect size due to the existence of heterogeneity in the general characteristics of the samples (e.g., sample size, gender, age and population context) [36]. The proportion of variation in the pooled estimates caused by heterogeneity was calculated with *I²* index. Subgroup analyses were performed on the possible conceptual moderator variables type of blockage (anticipated or unanticipated) and degree of goal blockage (low or high). Sensitivity analyses were used to investigate whether results were robust to study design (quasi-experimental or cross-sectional and longitudinal) and study quality (low, average or high). We used the *X2* test to assess differences in subgroup analyses. Finally, publication bias was checked with Egger’s test and by visual inspection of the funnel plots [36]. In the presence of publication bias, the adjustment of the pooled estimates was done by using the trim and fill test [37]. The analyses were performed with Comprehensive Meta-analysis software [38].

## Results

### Description of studies identification and selection procedure

**Fig 1:**
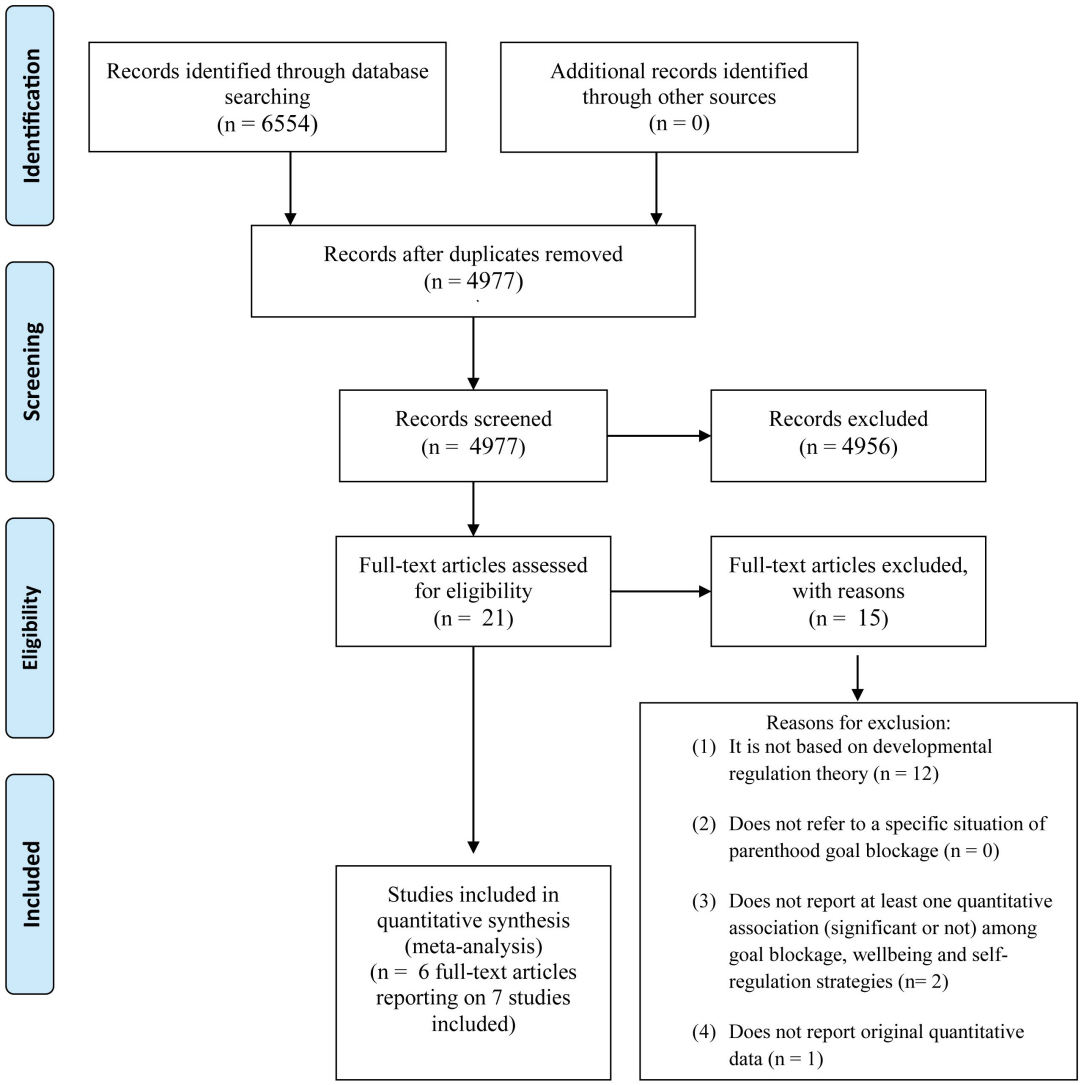
Flowchart of Studies Identification and Selection Procedure.

Fig 1 shows the results of the studies identification and selection procedure. As shown, a total of 4977 records were screened, 21 full texts assessed for eligibility, and six articles reporting on seven studies included in the meta-analyses [34, 39–43].

### General Characteristics of the Included Studies

Table 1 contains the general characteristics of the seven studies included. Two studies were conducted in the United States and five in Europe. A total of 672 people participated, with five out of seven studies (71%) sampling only women and 29% sampling only men. Two studies (29%) (Kraaij et al., [39] and Salmela-Aro & Suikkari [40]) included couples in the total number of men and women who participated. Four studies (57%) assessed people from the general population at different parenthood deadline phases and three out of seven studies (43%) sampled patients undergoing fertility treatment, and one sampled both. Two studies had a longitudinal design (29%), four were quasi-experimental (57%) and one had a cross-sectional design (14%). Table 2 shows the conceptualization and operationalization of the study variables. Three out of seven studies (43%) investigated the association between goal blockage and wellbeing and three of seven (43%) the associations between goal blockage and self-regulation strategies. Almost all the included studies (86%) analyzed the association between self-regulation strategies and wellbeing. Table 3 shows the measures used to assess goal blockage, wellbeing and self-regulation strategies and their reliability (when applicable). Three out of seven studies (43%) operationalized objective goal blockage as participant age, one out of seven (14%) used infertility diagnosis and three out of seven (43%) used subjective self-report items about perceived goal blockage. All the included studies assessed wellbeing with well-known and widely validated measures (e.g., Center for Epidemiology Studies Depression Scale). Six out of seven studies (86%) measured negative states of wellbeing and five of seven (71%) also measured positive states of wellbeing. In total, 100% studies assessed either negative or positive wellbeing.

**Table 1:** General Characteristics of the Studies.

Studies (N = 7)	Country	Sample N, gender, mean age (*SD*), age range	Sample characteristics (population and context)	General aim (as described by the authors)	Study Design
Heckhausen (2001) (study 1)	Germany	94 women, 27–46 years	General population– 2 groups: (1) women who passed parenthood deadline (40–46, no children) and (2) women who are currently approaching the deadline with no children (27–33, no children)	To explore self-regulation strategies (engagement and disengagement) individuals use in different stages of parenthood goal blockage.	Quasi-Experimental
Heckhausen (2001) (study 2)	Germany	126 women, 29–56 years	General population– 2 groups: (1) women who passed parenthood deadline (39–56, no children) and (2) women currently approaching the deadline (29–35, no children)	To explore self-regulation strategies (engagement and disengagement) individuals use in different stages of parenthood goal blockage.	Quasi-Experimental
Kraaij (2009)	Netherlands	59 women and 24 men, 45 (5.95) years	Infertile Patients	To explore associations between coping strategies, goal adjustment strategies (disengagement and reengagement) and positive and negative affect.	Cross-Sectional
Salmela-Aro (2008)	Finland	54 women, 33.92 (0.34) years and43 men, 35.68 (0.45) years	Infertile Patients	To examine child-related goal adjustment during infertility treatments and how it affects wellbeing.	Longitudinal
Thompson (2011)	USA	47 women, 33.13 (5.57) years	Infertile Patients	To examine associations between goal adjustment and psychological adjustment in the context of infertility.	Longitudinal
Light (2006)	USA	Urgent group: 29 women, 27.86 (2.33) years; Passed group: 28 women, 43.96 (3.09) years	General population—women who self-reported they had never had children	To investigate the attentional mechanisms related to the self-regulation strategies of goal engagement and disengagement in a lifespan context.	Quasi-Experimental
Kotter-Grühn (2009)	Germany	168 women^a^, 45.20 (6.60) years	General population and Infertile patients	To investigate whether an intense desire for ideal states of life (life longings) emerge when individuals are confronted with an unattainable goal and to investigate if pursuing an unattainable goal as a life longing leads to high wellbeing.	Quasi-Experimental

(USA) United States of America; (*SD*) = standard deviation. ^a^Self-regulation strategies of disengagement and reengagement were not assessed in 66 out of 168 women who indicated they did not have a former or current parenthood goal.

**Table 2:** Study Variables Conceptualization and Operationalization.

Studies	Theory	Goal blockage	Self-regulation strategies	Wellbeing	Associations investigated		
					Goal Blockage—Wellbeing	Goal Blockage—Self-Regulation	Self-Regulation—Wellbeing
Heckhausen (2001) (1)	The Action-phase Model of Developmental Regulation (Heckhausen, 1999)	Objective GB—Age	Indicators (content categories of relevant information) of goal disengagement and reengagement	Positive and negative affect	NI	Associations between indicators of goal disengagement and goal reengagement with goal blockage	Associations between indicators of goal disengagement and goal reengagement with positive and negative affect
Heckhausen (2001) (2)	The Action-phase Model of Developmental Regulation (Heckhausen, 1999)	Objective GB—Age	Goal disengagement	Depressive symptoms	NI	NI	Associations between goal disengagement and depressive symptoms
Kraaij (2009)	Theoretical assumptions of Adaptive Goal Adjustment (Wrosch et al., 2003)	Objective GB—men and women with infertility diagnosis	Goal disengagement and goal reengagement	Positive and negative affect	NI	NI	Associations between goal disengagement and goal reengagement with positive and negative affect
Salmela-Aro (2008)	Several different theoretical frameworks about child-related goal appraisals	Subjective GB—3 items, e.g. “How far has this goal progressed?”	NI	Depressive symptoms	Association between goal blockage and depressive symptoms	NI	NI
Thompson (2011)	Theoretical assumptions of Adaptive Goal Adjustment (Wrosch et al., 2003)	Subjective GB—1 item, “How blocked do you feel in your goal of becoming a parent?”	Goal disengagement and goal reengagement	Depressive symptoms and positive states of mind	Associations between goal blockage and depressive symptoms and goal blockage and positive states of mind	Associations between perceived goal blockage and goal disengagement and reengagement	Associations between goal disengagement and goal reengagement with depressive symptoms and positive states of mind
Light (2006)	The Action-phase Model of Developmental Regulation (Heckhausen, 1999)	Objective GB—Age	Indicators of goal disengagement (number of sentences recalled)	Positive and negative affect	NI	NI	Associations between goal disengagement and positive affect and goal disengagement and negative affect
Kotter-Grühn (2009)	Theoretical assumptions of Adaptive Goal Adjustment (Wrosch et al., 2003)	Subjective GB -6 items, e.g. “I am sure I can fulfill my wish for a child sometime.”	Goal disengagement and goal reengagement	Life satisfaction (Happiness)	Associations between goal blockage and life satisfaction	Associations between goal blockage and goal disengagement and goal blockage and goal reengagement	Associations between goal disengagement and life satisfaction and goal reengagement and life satisfaction

GB, goal blockage; NI, not investigated in the study.

**Table 3:** Measures used to assess Goal blockage, Self-regulation and Wellbeing and its Reliability (when applicable).

Studies	Goal blockage	*α*	Wellbeing	*α*	Self-regulation	*α*
Heckhausen (2001) (1)	Women age, 27–46 years	NA	Positive and Negative Affect Scale (PANAS; Watson, Clark & Tellegen, 1988)	NR	Open format questionnaire (Heckhausen, 1997)	92% inter-rater agreement
					Memory recall task	98% inter-rater agreement
Heckhausen (2001) (2)	Women age, 29–56 years	NA	Center for Epidemiology Studies Depression Scale (CES-D; Radloff, 1977)	.90	OPS Scale (four subscales) for Childwish developed by the authors	.87(SPCS),.83(SSCS),.87(CPCS),.39(CSCS)
Kraaij (2009)	Definitive infertility	NA	Positive and Negative Affect Scale (PANAS; Watson, Clark & Tellegen, 1988):		Goal adjustment scale (Wrosch et al., 2003):	
			Positive Affect Scale	.90	Goal Disengagement Scale	.71
			Negative Affect Scale	.84	Goal Reengagement Scale	.88
Salmela-Aro (2008)	3 self-report items about perceived goal attainability (e.g. “How far has this goal progressed?”)	♀(.63, .60, .67, .78, .70, .78); ♂ (.65, .86, .88, .86, .86, .64)	Beck Depression Inventory	♀(.92,.90); ♂ (.90, .89)	NI	NI
Thompson (2011)	1 self-report item about subjective goal blockage (e.g., “How blocked do you feel in your goal of becoming a parent?”)	NA	Center for Epidemiology Studies Depression Scale (CES-D; Radloff, 1977)	.94	Self-reported measure developed by the authors:	
			Positive States of Mind Scale (Horowitz et al., 1988)	.92	Goal Disengagement Scale	.92 (Time 1); .89 (Time 2)
					Goal Reengagement Scale	.89 (Time 1); .87 (Time 2)
Light (2006)	Women age:		Positive and Negative Affect Scale (PANAS; Watson, Clark & Tellegen, 1988):		Memory recall task	97% inter-rater agreement
	Urgent group: 27.86 (2.33) years	NA	Positive Affect Scale	.83		
	Passed group: 43.96 (3.09) years	NA	Negative Affect Scale	.78		
Kotter-Grühn (2009)	Subjective attainability 6-item scale partly taken from the Life Longing Realization Scale (Scheibe, 2005)	.81	The Temporal Satisfaction with life Scale (Pavot, Diener & Suh, 1998)	.89	Goal adjustment scale (Wrosch et al., 2003):	
					Goal Disengagement Scale	.84
					Goal Reengagement Scale	.95

*α*, Cronbach’s alpha of the present studies samples; NR, not reported in the study; NI, not investigated; NA, non-applicable; SPCS, Selective primary control subscale; SSCS, Selective secondary control subscale; CPCS, Compensatory primary control subscale; CSCS, compensatory secondary control subscale; GDS, goal disengagement scale; GRE, goal reengagement scale; ♀, women results; ♂, men results.

### Quality assessment

Supplemental information in S3 Table shows the quality rating details for all the studies included in the present systematic review and meta-analyses. From the total number of studies included, none met all the representativeness criteria, 100% had an adequate use of a theoretical framework, 14% (n = 1) met all the measurement criteria for reliability, and none met the criteria for the evaluation of the hypotheses. The overall quality ratings indicate four average (57%) and three poor (43%) quality studies with almost perfect agreement (S.M.S. and S.G., Cohen’s *k* = .89, *p* < .001).

### Meta-Analyses

**Fig 2:**
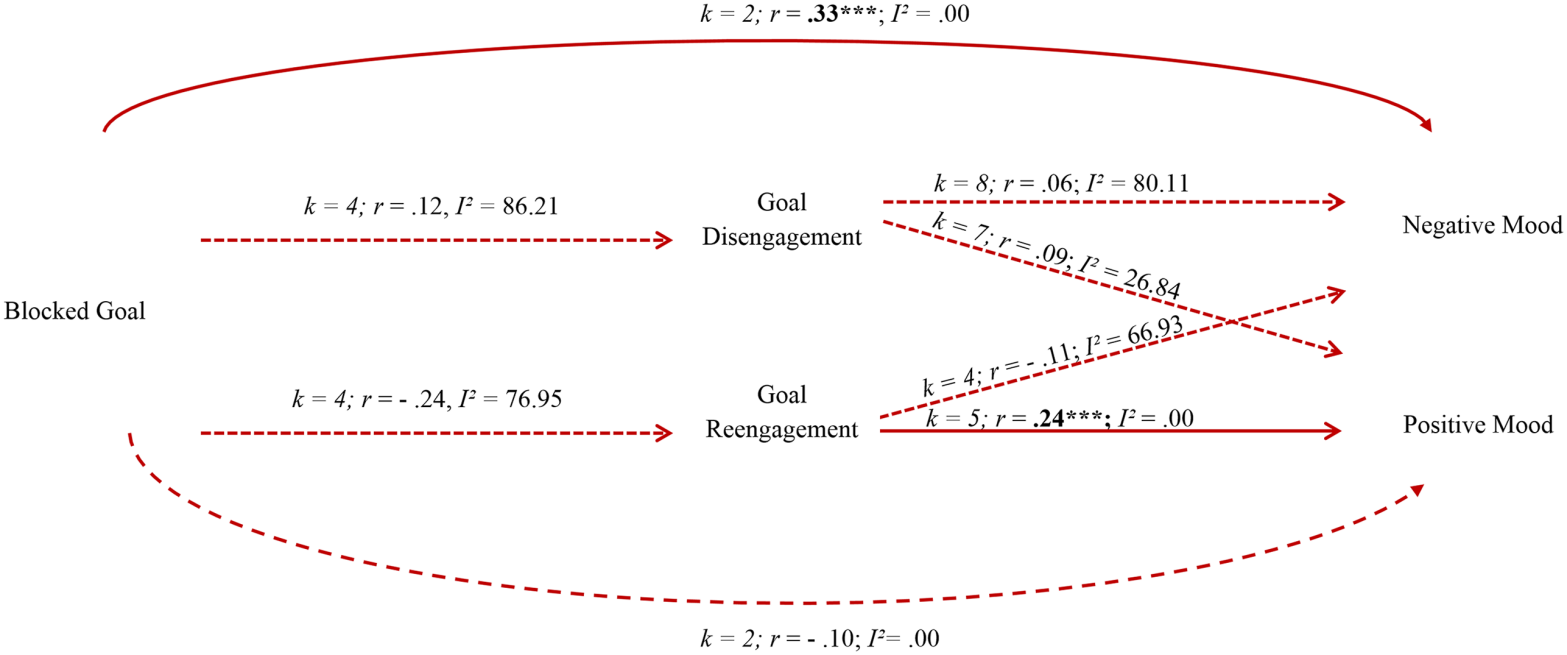
Summary Statistics for Random Effects Model of Pooled Effect Sizes. **[Legend]**This is not a structural equation model. Diagram reflects the proposed paths in developmental regulation theory. Lines refer to proposed associations tested in the meta-analyses. Continuous line = significant association; Dashed line = non-significant association; *k* = number of studies testing the association; *r* = correlation coefficient; *I^2^* = I squared index. ***p <. 001.

The diagram in Fig 2 presents a summary of the theoretically proposed hypotheses on which meta-analyses results have been mapped. Supplemental data in S4–S6 Tables show the effect size for the individual studies. Fig 2 shows that two of the eight associations were significant. Specifically, higher goal blockage was related to higher negative mood (*r* = .33, *p* < .001, *I² =* .00), and higher goal reengagement in alternative life goals was associated with higher positive mood (*r* = .24, *p* < .001, *I² =* .00).

### Moderation of effect size

Due to a lack of studies, it was not possible to perform subgroup analyses for the associations between blocked goal with wellbeing, and blocked goal with self-regulation strategies. The sensitivity analyses performed on the associations between blocked goal and self-regulation strategies were non-applicable for the variable study design, and no differences were found for the variable study quality (blocked goal with goal disengagement, *X*^*2*^ = 0.195, *p* = .66; blocked goal with goal reengagement, *X*^*2*^ = 2.425, *p* = .12). The subgroup and sensitivity analyses performed for the association between self-regulation strategies and negative mood are presented in S7 Table. Only two of the sixteen subgroup and sensitivity analyses were significant. The type of goal blockage moderated the association between goal disengagement and negative mood in a subgroup analysis. There was a significant difference between the anticipated and unanticipated subgroups (*X*^*2*^ = 4.867, *p* = .03) but individually (i.e., in each subgroup), these associations were non-significant (anticipated, *r* = .18, [-.04, .38], *p* = .11; non-anticipated, *r* = -.29, [-.58, .07], *p* = .11). The sensitivity analysis also showed that the direction of the association between goal disengagement and negative mood varied according to study quality (*X*^*2*^ = 4.867, *p* = .03). However, within each group these associations were non-significant.

Supplemental data in S8 Table shows the moderation and sensitivity analysis performed on the association between self-regulation strategies (goal disengagement and goal reengagement) and positive mood. There was not any significant evidence for self-regulation strategies and positive mood in the subgroup or sensitive analysis performed.

### Publication Bias

Visual inspection of the funnel plot and the Egger’s test indicated the presence of publication bias for the associations between blocked parenthood goal and goal reengagement (*intercept* = - 9.28, *t =* 7.97, *p =* .01) and between goal disengagement and negative mood (*intercept* = 8.65, *t* = 3.37, *p =* .01). Trim and fill identified one study missing to the right of the mean for the association between goal blockage and goal reengagement (i.e., positive association, see figure in S1 Fig) (adjusted value, *r* = -.17 [- .40, .08]) and none missing for the association between goal disengagement and negative mood. The presence of publication bias was not detected by Egger’s test for the association between goal disengagement and positive mood but was identified by visual inspection of the funnel plot and the trim and fill test suggesting the existence of one study missing to the right of the mean (i.e., positive association, see figure in S2 Fig) (adjusted value, *r* = .12 [-.00, .24]).

## Discussion

### Main Findings

The results of the meta-analyses indicate that the existing evidence regarding a blocked parenthood goal only partially supported two of the three main predictions of developmental regulation theories. Overall, people were, as predicted, more likely to experience poorer wellbeing (e.g., higher depression, negative affect) when facing a blocked parenthood goal but were not more likely to disengage from the parenthood goal and reengage in alternative life goals. Further, only reengagement showed to be positively associated with wellbeing. However, some subgroup differences found suggest that the predictions may be valid but need to be reconsidered in light of other moderating influences relevant to parenthood goals. The main limitations of the review were the scarce body of primary research on parenthood goal blockage, self-regulation and wellbeing and the presence of publication bias. The main limitation of the primary research was the lack of consistent operational definitions for blocked parenthood goal. We will develop each of these issues further.

The meta-analyses did not provide strong support for developmental regulation theories for a blocked parenthood goal. There may be multiple explanations for poor theoretical fit. The unique features of this goal may mean that developmental regulation is less able to account for relevant outcomes. First, its dyadic nature implies the existence of a shared investment by both members of a couple [44]. Past research on developmental regulation has almost always taken the individual level of self-regulation as the main unit of analysis, and this was the case for primary studies included in the present meta-analyses. More recent studies suggest that the use of an individual level of analysis weakens the explanatory power of developmental regulation theories by misrepresenting the phenomenon of self-regulation [44]. This is because by only capturing the individual level of regulation, researchers are neglecting the influence of interpersonal processes that have been shown to affect the way individuals regulate in relation to personal goals such as losing weight [45, 46].

An alternative perspective is that developmental regulation theory is relevant to the blocked parenthood goal but the theory needs to be reconsidered in light of moderator influences on key constructs that arise from this unique goal. First, goal reengagement was associated with positive (life satisfaction, positive frame of mind) but not negative (depressive symptoms, negative affect) wellbeing, indicating that reengagement produces a divergent effect in wellbeing: it makes people feel good but does not necessarily take away the stress, disappointment or discontent that comes from failing to achieve an important life goal. Previous studies found that reengaging in other life goals when facing an unattainable goal was associated with low levels of depression [6] but these were not observed in the present study in relation to negative mood. Divergence may be specific to the parenthood goal because it is a hard goal to replace—one cannot, like with other goals, easily replace it. A possible alternative is to substitute biological parenthood with adoption, but it does not suit everyone and tends to delay adjustment [47]. If parenthood cannot be easily replaced, then one must consider that the purpose of reengagement is coping with the stress caused by the parenthood loss rather than exploring a way forward after goal failure. Another possible explanation is that the impact of goal reengagement on wellbeing is dependent on the ability to disengage from the parenthood goal. In a study conducted with undergraduate students, failing to reengage led to high perceived stress and low self-mastery levels and this happened especially for those students struggling to disengage from an unattainable goal [6]. This also suggests that the effects of disengagement and reengagement on wellbeing are not independent but likely to interact, an important issue to be addressed in future research. Simultaneously, goal reengagement was not related to better wellbeing in young adults who were disengaged from an unattainable goal and this was possibly because of more optimistic expectations about the future and the perception of more opportunities that they could engage with later on [6]. Given the participants in the primary studies mostly included young and middle aged adults, it can be that participants still had positive expectations about achieving parenthood in the future.

A second form of moderation that should be considered in theoretical work is whether the way in which goals become blocked matters to eventual outcomes. We found that the value of disengagement differs according to whether goals were anticipated or not. The data suggests that, in accordance with the theory, it is adaptive to disengage from unanticipated goals (infertility). However, it showed that it is maladaptive to disengage from anticipated goals (age). Decision-making research may be useful to explain this unexpected result. It suggests that when individuals are confronted with a goal that implies anticipated action (i.e., anticipated blockage), they perceive themselves to be responsible for the consequences of engaging or not with the goal [48]. It is this perception of responsibility, which is absent in unanticipated goals, that may be the reason for worse adjustment. Indeed, research showed that it manifests in terms of anticipated regret, which corresponds to a negative emotional state that occurs when people anticipate the consequences of their decision-making [48, 49]. Therefore, the more one disengages by delaying decision-making, the more one delays goal achievement and, consequently, experiences more regret. However, it should be noted that although the subgroup comparisons were significant, the omnibus subgroup tests were not, due to the small sample size. For that reason, this finding needs replication in future research.

Although parenthood is one of the most central goals in adulthood there is still a scarce body of research on developmental regulation theories and parenthood goal. Of the 4977 records screened in our review, only 7 studies examined parenthood goal blockage from the perspective of developmental regulation theories. 12 studies were excluded because they used other theoretical frameworks such as coping theories [50–52]. This was done to ensure the conceptual validity of the present study. Indeed, coping and developmental regulation strategies are not conceptually equivalent. For instance, while avoidant coping and disengagement imply withdrawing efforts to address the stressor/blockage, they differ in their functional value. The first implies non-confrontation while the second implies acceptance regarding the stressor/blockage. Therefore they are also expected to have differential impact on wellbeing.

The review also highlighted a lack of conceptual clarity in the way that the blocked parenthood goal has been operationalized in research. This should be addressed by future research. In the medical literature, people are considered to have fertility problems when they have had regular, unprotected sexual intercourse for 12 or more months without conceiving [27] or have a pre-defined medical problem (e.g., Turner syndrome). This level of precision should be used to define a medical unanticipated parenthood goal blockage within the developmental regulation literature. Further, in the medical literature the cut-off for old parenthood age tends to be 35 years [53]. Applying the same logic, women and couples should be said to be facing an anticipated parenthood goal blockage when women are 35 or older. A similar cut-off needs to be identified for single or gay men pursuing parenthood. Defining a goal blockage using subjective indicators is a more complex issue. First, different scales are currently being used. From the ones included in this review, the subjective attainability 6-item scale used by Kotter-Grühn et al. [43] was the most reliable (*α* = .81) but its validity is not known. Second, regardless of the scale used, it would be important to try to establish cut-off scores that would indicate a subjective understanding of when a goal is blocked. Different methods have been developed that can be used to achieve this. For instance, the World Health Organization advises the use of consistent magnitudes between the different labels of the response scales [54], e.g., finding the best textual descriptors that represent regular intervals in terms of the degree of blockage such as halfway between ‘not blocked’ and ‘completely blocked’. Agreement on a precise operational definition of a blocked parenthood goal in the literature could help solve the problem of the lack of conceptual clarity.

The quality of the primary research included was variable. Of the seven studies, 43% were rated as low quality, mainly because the studies’ representativeness could not be assessed (due to lack of reporting of response rates), poor quasi-experimental and cross-sectional designs, and inadequate testing of their research hypotheses (e.g., low statistical power). Past research on developmental regulation has highlighted the importance of using longitudinal designs when testing developmental regulation theories in order to capture the micro-sequential changes of self-regulation strategies [4]. However, the decision about whether and when to have children is one that unfolds over a long period of time, and identifying a practical research paradigm that can allow for an acceptable and feasible longitudinal study of these constructs in couples about to start trying to conceive will be a challenge. The use of clinical samples of patients undergoing treatment is practical, but also has its challenges. For instance, an important one is that only about 56% of people with fertility problems opt to seek medical advice [55], which means that samples would not necessarily represent the population of people experiencing an unanticipated goal blockage. The sensitivity analysis performed on the association between goal disengagement and negative mood suggested non-consistency in findings reported according to study quality. This analysis showed that when only average quality studies were included in the meta-analysis, greater goal disengagement was associated with lower negative mood, supporting the theoretical prediction. However, even if subgroup comparisons were significant, due to small sample size, the omnibus subgroup tests were non-significant and this finding needs further clarification from future research. In sum, sensitivity analyses suggest that results may vary according to the quality of the studies included, but unfortunately there were no high quality studies in primary research to make an estimation of what results would be. Definitive conclusions about the applicability of developmental regulation theory to the parenthood goal should therefore be made when relevant research achieves a higher quality standard.

Finally, low power is likely to have undermined the comprehensiveness of the evidence base. The presence of publication bias in two of the associations tested indicated that the ‘missing’ studies were those that would support developmental regulation theory. The absence of such studies is unlikely to be a bias due to preference for other explanatory theoretical approaches such as cognitive coping theories to explain delayed or blocked parenthood goals. Most likely, bias is due to the absence of studies capable of showing a significant effect as more than half of the studies did not have enough power to detect significant associations between the study variables.

### Strengths and Limitations

#### Meta-analyses

This meta-analysis is timely and appropriate given the lack of systematic reviews evaluating developmental regulation theories. Indeed, although two comprehensive and integrative reviews on this topic exist [4, 56], these did not involve an exhaustive and systematic review of the literature and quantitative evaluation of basic predictions. The present meta-analyses followed a systematic implementation procedure, and official guidelines for conducting systematic reviews and meta-analysis (i.e., PRISMA statement). The search strategy used was systematic and exhaustive as it covered eight databases. The processes of study selection and assessment were done by two independent researchers (S.M.S. and S.G.) and based on detailed *a priori* defined criteria. Subgroup analyses were pre-specified before being carried out in order to include relevant conceptual moderator variables that could contribute to the progress of the developmental regulation field. In addition, sensitivity analyses were performed to assess the robustness of results in relation to methodological issues and the risk of publication bias was controlled by using different statistical tests (such as Egger’s test and Trim and fill). All these contributed to an extensive test of the reliability of results.

#### Studies Included

There are important limitations related to the primary research that also influenced the present meta-analyses. First, all studies were conducted in developed countries in Europe and also in the USA, which means that the results reported are influenced by the specific values and norms related to the parenthood goal in those cultures. Some studies have shown that the motivational processes of parenthood are influenced by the individuals’ cultural values and norms [57]. Furthermore, regarding the sample characteristics of the studies included, all of them investigated heterosexual couples and did not focus on other possible populations, such as gay couples that usually have to deal with higher levels of parenthood goal blockage due to social norms and values [58].

In sum, the present systematic review and meta-analyses brings forward a complex interplay between blocked parenthood goal, self-regulation strategies and wellbeing which has not yet been address by the empirical research available so far. Developmental regulation theories have been supported in different contexts of life-goal blockages, such as intimate relationships and health conditions [12,13]. However, based on our results it seems premature to make any confident conclusion about the validity of developmental regulation theories when applied to the specific situation of parenthood goal blockage. A contribution to the developmental regulation field in the specific context of a blocked parenthood goal has been made by proposing a clear conceptualization of blocked parenthood goal based on evidence from past research. Further studies are required to evaluate the value of developmental regulation theories for parenthood goal because too few studies exist, and important conceptual issues and methodological limitations have not yet been resolved in primary research.

## Supporting Information

S1 Fig Publication Bias Funnel Plot for the Association between Goal Blockage and Goal Reengagement. (TIF)

S2 Fig Publication Bias Funnel Plot for the Association between Goal Disengagement and Positive Mood. (TIF)

S1 Table Full search strategy. (PDF)

S2 Table List of excluded studies and reasons for exclusion. Reasons for exclusion classified as: (1) It is not based on developmental regulation theory; (2) Does not refer to the specific situation of parenthood goal blockage; (3) Does not report at least one quantitative association (significant or not) among goal blockage, wellbeing and self-regulation strategies; (4) Does not report original quantitative data.
(PDF)

S3 Table Score details of Quality Assessment of Studies. FU, Follow-up; SR, Self-Regulation; GB, Goal Blockage; WB, Wellbeing; NA, Not Applicable; NM, Not Mentioned; Quality ratings were grouped into low (0–4), average (5–9) and high (10–11).
(DOCX)

S4 Table Random Effect Model Results of Associations between Goal Blockage and Wellbeing and Goal Blockage and Self-regulation Strategies. *N*, sample size; *r*, correlation coefficient; *CI*, Confidence Interval; *LL*, lower limit; *UL*, upper limit; *p*, significance level; NI, not investigated in the study; ^a^Group labels verbatim from studies; Urgent group, group of women approaching parenthood deadline; Passed group, group of women who missed parenthood deadline; ***p <. 001.
(DOCX)

S5 Table Random Effect Model Results of Associations between Self-regulation Strategies and Negative Mood. *N*, sample size; *r*, correlation coefficient; *CI*, Confidence Interval; *LL*, lower limit; *UL*, upper limit; *p*, significance level; NI, not investigated in the study; ^a^Group labels verbatim from studies; Urgent group, group of women approaching parenthood deadline; Passed group, group of women who missed parenthood deadline.
(DOCX)

S6 Table Random Effect Model Results of Associations between Self-regulation Strategies and Positive Mood. *N*, sample size; *r*, correlation coefficient; *CI*, Confidence Interval; *LL*, lower limit; *UL*, upper limit; *p*, significance level; NI, not investigated in the study; ^a^Group labels verbatim from studies; Urgent group, group of women approaching parenthood deadline; Passed group, group of women who missed parenthood deadline;***p < .001.
(DOCX)

S7 Table Associations between Self-regulation and Negative Mood according to Subgroup and Sensitivity Analysis. *k*, number of studies; *r*, correlation coefficient; *CI*, confidence Interval; *LL*, lower limit; *UL*, upper limit; *X*^*2*^, chi-square; NA, not applicable because at least one of the groups only has one or no study; *p < .05. ^a^Cross-sectional and quasi-experimental studies were included in the same category in sensitivity analysis since both provide none or little evidence to infer causality. ^b^The quality of a study was categorized in the analyses as low, average or high according to the score obtained in the quality assessment.
(DOCX)

S8 Table Associations between Self-regulation and Positive Mood according to Subgroup and Sensitivity Analysis. *k*, number of studies; *r*, correlation coefficient; *CI*, Confidence Interval; *LL*, lower limit; *UL*, upper limit; *X*^*2*^, chi-square; NA, not applicable because at least one of the groups only has one or no study. ^a^Cross-sectional and quasi-experimental studies were included in the same category in sensitivity analysis since both provide none or little evidence to infer causality. ^b^The quality of a study was categorized in the analyses as low, average or high according to the score obtained in the quality assessment.
(DOCX)

S9 Table PRISMA Checklist. (DOC)
