# Supplementary figures and images for: Self-Regulation and Wellbeing When Facing a Blocked Parenthood Goal: A Systematic Review and Meta-Analysis
Source: PLoS One. 2016 Jun 23;11(6):e0157649. doi: 10.1371/journal.pone.0157649 (PMC4919102; doi:10.1371/journal.pone.0157649)

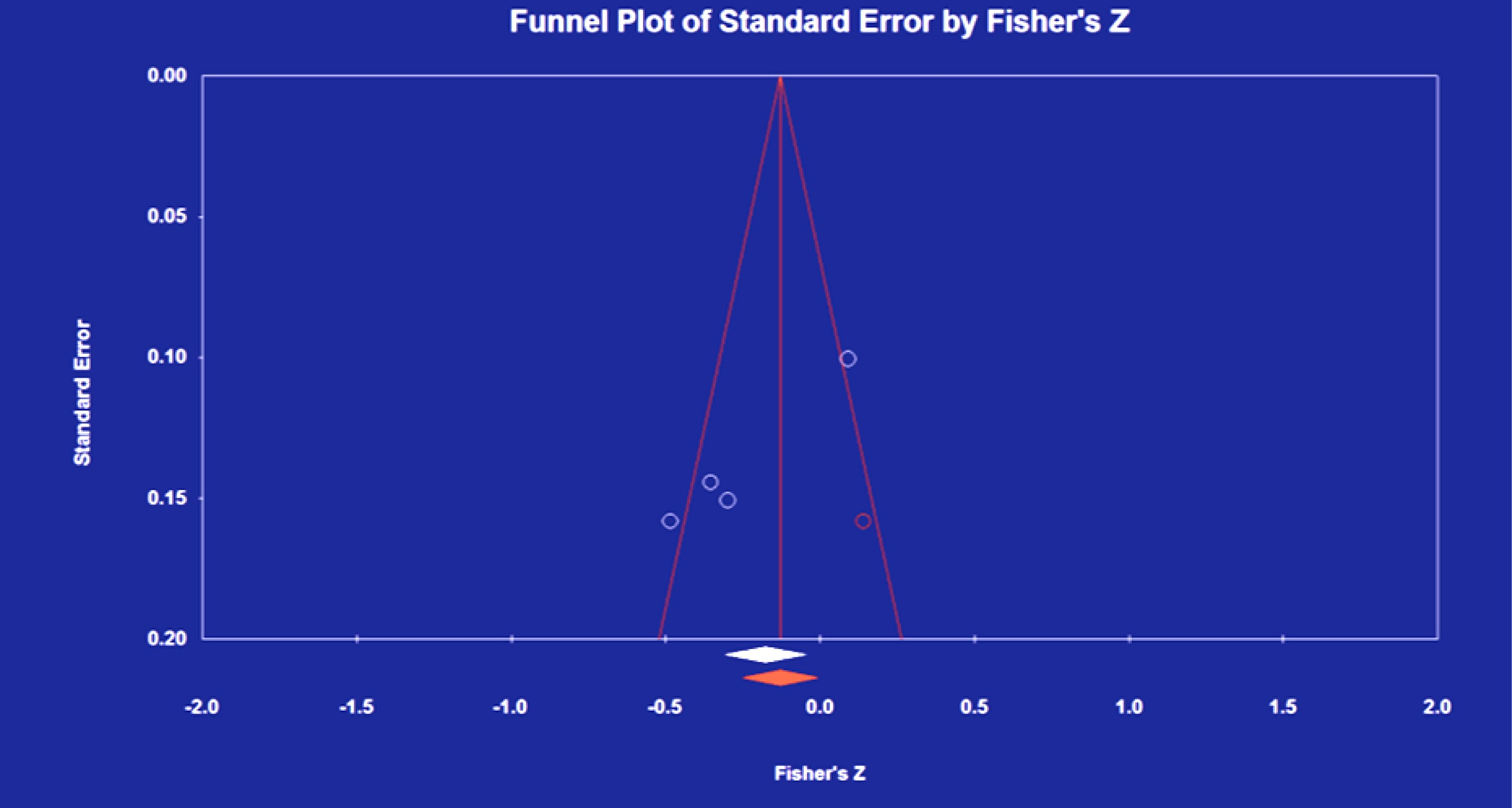

Supplement: S1 Fig — (TIF) [file pone.0157649.s001.tif]

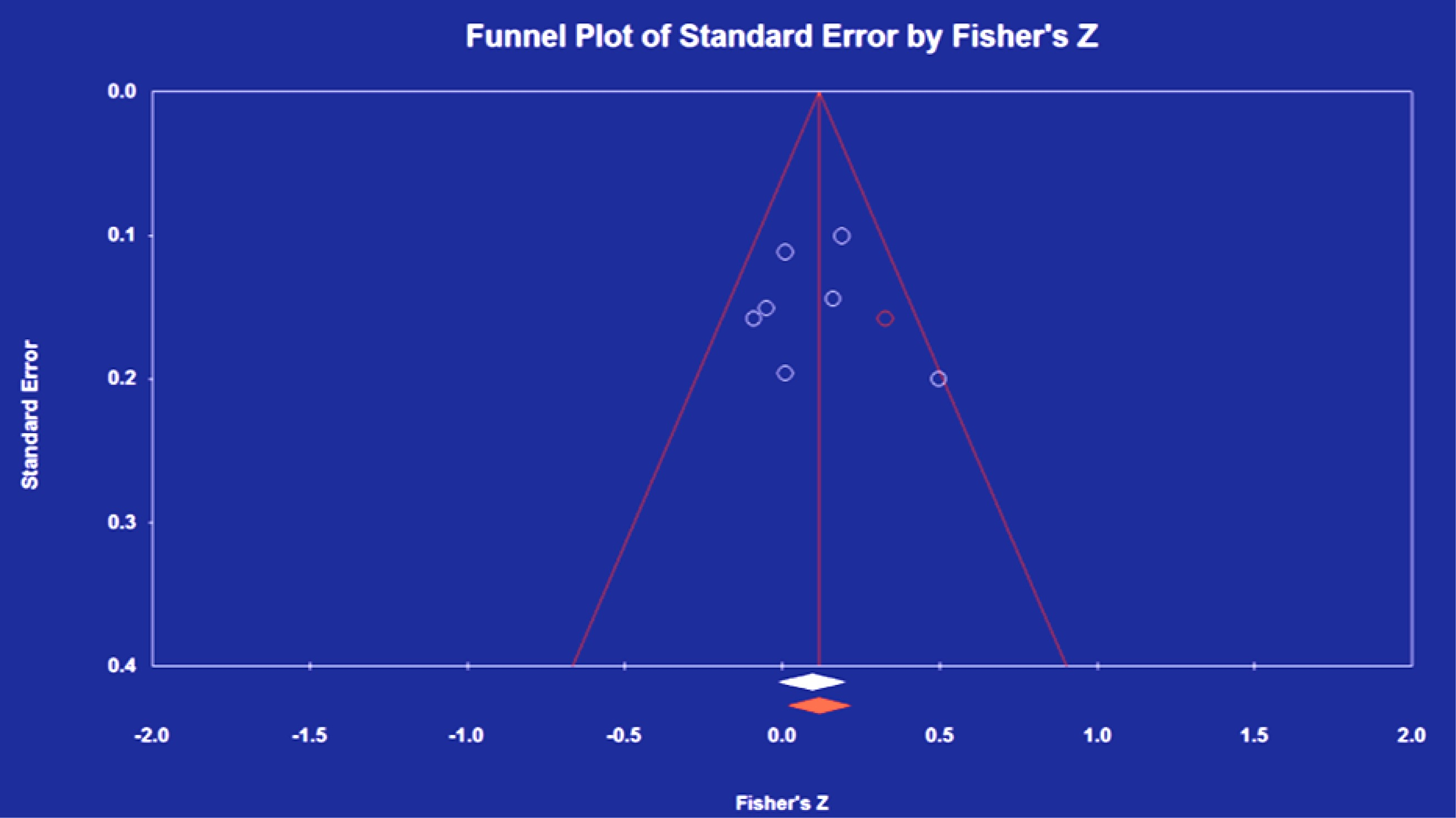

Supplement: S2 Fig — (TIF) [file pone.0157649.s002.tif]
