# Supplementary material for: Self-Regulation and Wellbeing When Facing a Blocked Parenthood Goal: A Systematic Review and Meta-Analysis
Source: PLoS One. 2016 Jun 23;11(6):e0157649. doi: 10.1371/journal.pone.0157649 (PMC4919102; doi:10.1371/journal.pone.0157649)
Supplement: S1 Table — (PDF) [file pone.0157649.s003.pdf]

	Search Term	Number of records
1	(block$ or unattain$ or unachiev$ or impossib$ or inaccessib$ or unreach$ or unfeasib$ or deadline or fail$ or "life transition" or infertil$ or steril$ or subfert$ or childless$).ab,ti.	3816344
2	((goal adj5 (parent$ or mother$ or maternity$ or fertil$ or pregnan$ or child$ or gestat$ or conceiv$ or conception or birth)) or (aim adj5 (parent$ or mother$ or maternity$ or fertil$ or pregnan$ or child$ or gestat$ or conceiv$ or conception or birth)) or (objective adj5 (parent$ or mother$ or maternity$ or fertil$ or pregnan$ or child$ or gestat$ or conceiv$ or conception or birth)) or (reach adj5 (parent$ or mother$ or maternity$ or fertil$ or pregnan$ or child$ or gestat$ or conceiv$ or conception or birth)) or (task adj5 (parent$ or mother$ or maternity$ or fertil$ or pregnan$ or child$ or gestat$ or conceiv$ or conception or birth)) or (achiev$ adj5 (parent$ or mother$ or maternity$ or fertil$ or pregnan$ or child$ or gestat$ or conceiv$ or conception or birth)) or (try$ adj5 (parent$ or mother$ or maternity$ or fertil$ or pregnan$ or child$ or gestat$ or conceiv$ or conception or birth)) or (pursui$ adj5 (parent$ or mother$ or maternity$ or fertil$ or pregnan$ or child$ or gestat$ or conceiv$ or conception or birth)) or (attempt$ adj5 (parent$ or mother$ or maternity$ or fertil$ or pregnan$ or child$ or gestat$ or conceiv$ or conception or birth)) or childbearing).ab,ti.	167482
3	(wellbeing or well-being or "mental health" or mental-health or "quality of life" or quality-of-life or anxi$ or depress$ or stress$ or adjust$ or distress$ or fulfil$ or satisf$ or meaning).ab,ti.	5318355
4	1 and 2 and 3	3947
5	(block$ or unattain$ or unachiev$ or impossib$ or inaccessib$ or unreach$ or unfeasib$ or deadline or fail$ or "life transition" or infertil$ or steril$ or subfert$ or childless$).ab,ti.	3816344
6	((goal adj5 (parent$ or mother$ or maternity$ or fertil$ or pregnan$ or child$ or gestat$ or conceiv$ or conception or birth)) or (aim adj5 (parent$ or mother$ or maternity$ or fertil$ or pregnan$ or child$ or gestat$ or conceiv$ or conception or birth)) or (objective adj5 (parent$ or mother$ or maternity$ or fertil$ or pregnan$ or child$ or gestat$ or conceiv$ or conception or birth)) or (reach adj5 (parent$ or mother$ or maternity$ or fertil$ or pregnan$ or child$ or gestat$ or conceiv$ or conception or birth)) or (task adj5 (parent$ or mother$ or maternity$ or fertil$ or pregnan$ or child$ or gestat$ or conceiv$ or conception or birth)) or (achiev$ adj5 (parent$ or mother$ or maternity$ or fertil$ or pregnan$ or child$ or gestat$ or conceiv$ or conception or birth)) or (try$ adj5 (parent$ or mother$ or maternity$ or fertil$ or pregnan$ or child$ or gestat$ or conceiv$ or conception or birth)) or (pursui$ adj5 (parent$ or mother$ or maternity$ or fertil$ or pregnan$ or child$ or gestat$ or conceiv$ or conception or birth)) or (attempt$ adj5 (parent$ or mother$ or maternity$ or fertil$ or pregnan$ or child$ or gestat$ or conceiv$ or conception or birth)) or childbearing).ab,ti.	167482
7	(engag$ or disengag$ or "letting go" or "let go" or "give up" or "giving up" or "gave up" or "stop try" or "stop trying" or "stopped trying" or reengag$ or adjust$ or "primary control" or "secondary control" or assimilat$ or accomodat$ or select$ or optimiz$ or compens$ or attain$ or regulat$ or channeling or choice or co-agency or co-regulat$).ab,ti.	8583615
8	5 and 6 and 7	5361
9	4 or 8	7628
10	limit 9 to humans	6554
