# Supplementary material for: Self-Regulation and Wellbeing When Facing a Blocked Parenthood Goal: A Systematic Review and Meta-Analysis
Source: PLoS One. 2016 Jun 23;11(6):e0157649. doi: 10.1371/journal.pone.0157649 (PMC4919102; doi:10.1371/journal.pone.0157649)
Supplement: S2 Table — Reasons for exclusion classified as: (1) It is not based on developmental regulation theory; (2) Does not refer to the specific situation of parenthood goal blockage; (3) Does not report at least one quantitative association (significant or not) among goal blockage, wellbeing and self-regulation strategies; (4) Does not report original quantitative data. (PDF) [file pone.0157649.s004.pdf]

Manuscript	Reason*
Fisher JRW, Baker GHW, Hammarberg K. Long-term health, well-being, life satisfaction, and attitudes toward parenthood in men diagnosed as infertile: challenges to gender stereotypes and implications for practice. Fertil Steril. 2010 Jul;94(2):574-80. Epub 2009 Apr 1. doi: 10.1016/j.fertnstert.2009.01.165.	1
Baor L, Blickstein I. (2005). En route to an "instant family": Psychosocial considerations.Obstet Gynecol Clin North Am. 2005 Mar;32(1):127-39. PMID:15644294	4
Monga M, Alexandrescu B, Katz SE, Stein M, Ganiats T. Impact of infertility on quality of life, marital adjustment, and sexual function. Urology. 2004 Jan;63(1):126-30. PMID:14751363	1
Callan VJ, Kloske B, Kashima Y, Hennessey JF. Toward understanding women's decisions to continue or stop in vitro fertilization: The role of social, psychological, and background factors. J In Vitro Fert Embryo Transf. 1988 Dec;5(6):363-9. PMID: 3221127	1
Daniluk, JC (2001). Reconstructing their lives: A longitudinal, qualitative analysis of the transition to biological childlessness for infertile couples. Journal of Counseling & Development. 2001 Fall;79(4): 439-49. doi:10.1002/j.1556-6676.2001.tb01991.	1
Terry, DJ, Hynes, GJ. Adjustment to a Low-Control Situation: Reexamining the Role of Coping Responses. J Pers Soc Psychol. 1998;74(4):1078-1092. doi:.1037/0022-3514.74.4.1078	1
Wu AK, Elliott P, Katz PP, Smith JF. Time costs of fertility care: The hidden hardship of building a family. Fertil Steril. 2013 Jun;99(7):2025-30. Epub 2013 Feb 28. doi: 10.1016/j.fertnstert.2013.01.145.	1
Moura-Ramos M, Gameiro S, Soares I, Santos TA, & Canavarro MC. Does infertility history matter? Complex effects of infertility history in psychological adjustment during assisted reproductive treatments. Proceedings of the 27th Annual Meeting of the European Society of Human Reproduction and Embryology; 2011 Jul 3-6; Stockholm, Sweden. Hum. Reprod: 26 (suppl 1): i78-i80. doi: 10.1093/humrep/26.s1.52	1
McQueeney DA, Stanton AL, Sigmon S. Efficacy of emotion-focused and problem-focused group therapies for women with fertility problems. J Behav Med. 1997 Aug;20(4):313-31. PMID: 9298432	1
Freeman EW, Rickels K, Tausig J, Boxer A, Mastroianni L Jr, Tureck RW. Emotional and psychosocial factors in follow-up of women after IVF-ET treatment. A pilot investigation. Acta Obstet Gynecol Scand. 1987;66(6):517-21. PMID:3425253	1
McLaney MA, Tennen H, Affleck G, Fitzgerald T. Reactions to impaired fertility: the vicissitudes of primary and secondary control appraisals. Womens Health. 1995 Summer;1(2):143-59. PMID: 9373377	1
Clark LF, Henry SM, Taylor, DM. Cognitive examination of motivation for childbearing as a factor in adjustment to infertility. In: Stanton AL, Dunkel-Schetter C, editors. Infertility: Perspectives from stress and coping research. New York: Springer Science+Business Media New York; 1991. p.157-80.	1
Benyamini Y. Hope and fantasy among women coping with infertility and its treatments. In: Jacoby R, Keinan J, editors. Between stress and hope: from a disease-centered to a health-centered perspective. US: Praeger Publishers; 2003. p.141-60.	3
Tennen H, Affleck G, Mendola R. Causal explanations for infertility: Their relation to control appraisals and psychological adjustment.In: Stanton AL, Dunkel-Schetter C, editors. Infertility: Perspectives from stress and coping research. New York: Springer Science+Business Media New York; 1991. p.109-31.	1
Thompson EH, Woodward JT, Stanton AL. Dyadic Goal Appraisal During Treatment for Infertility: How Do Different Perspectives Relate to Partners' Adjustment?. Int J Behav Med. 2012 Sep;19(3):252-9. doi: 10.1007/s12529-011-9172-7.	3
