# Supplementary material for: Self-Regulation and Wellbeing When Facing a Blocked Parenthood Goal: A Systematic Review and Meta-Analysis
Source: PLoS One. 2016 Jun 23;11(6):e0157649. doi: 10.1371/journal.pone.0157649 (PMC4919102; doi:10.1371/journal.pone.0157649)
Supplement: S3 Table — FU, Follow-up; SR, Self-Regulation; GB, Goal Blockage; WB, Wellbeing; NA, Not Applicable; NM, Not Mentioned; Quality ratings were grouped into low (0–4), average (5–9) and high (10–11). (DOCX) [file pone.0157649.s005.docx]

|  | **Representativeness of the study sample**  **(0-4 points)** | **Adequate theoretical framework**  **(0-1 point)** | **Measurement**  **(0-2 points)** | **Evaluation of the hypotheses**  **(0-4 points)** | **Overall quality**  **(0-11)** |
| --- | --- | --- | --- | --- | --- |
| Heckhausen (2001) (1) | - sample included experiencing a blockage to parenthood goal (0)  - response rate (NM - 0)  - FU response rate (NA)  - FU participants compared with baseline participants (NA) | - Aims and hypotheses clearly derived from the Action-phase Model of Developmental Regulation (1) | - validated measures used  (SR – no; GB – NA; WB – yes) (0.5)  -reliable measures used  (SR – yes; GB –NA; WB – no) (0.5) | - causal relationship between variables assessed (0)  - enough power to detect significant associations (0)  - all possible confounders assessed (0)  - statistics analysis plan (0) | 2  (low) |
| Heckhausen (2001) (2) | - sample included experiencing a blockage to parenthood goal (0)  - response rate (NM - 0)  - FU response rate (NA)  - FU participants compared with baseline participants (NA) | - Aims and hypotheses clearly derived from the Action-phase Model of Developmental Regulation (1) | - validated measures used  (SR – yes; GB – NA; WB – yes) (1)  - reliable measures used  (SR – yes; GB – NA; WB – yes) (1) | - causal relationship between variables assessed (0)  - enough power to detect significant associations (0)  - all possible confounders assessed (0)  - statistics analysis plan (0) | 3  (low) |
| Kraaij (2009) | - sample included experiencing a blockage to parenthood goal (1)  - response rate (NM - 0)  - FU response rate (NA)  - FU participants compared with baseline participants (NA) | - Aims and hypotheses clearly derived from the theoretical assumptions of Adaptive Goal Adjustment (1) | - validated measures used  (SR – yes; GB – NA; WB – yes) (1)  - reliable measures used  (SR – yes; GB – NA; WB – yes) (1) | - causal relationship between variables assessed (0)  - enough power to detect significant associations (0)  - all possible confounders assessed (0)  - statistics analysis plan (1) | 5  (average) |
| Salmela-Aro (2008) | - sample included experiencing a blockage to parenthood goal (1)  - response rate (NM - 0)  - FU response rate (55% - 0)  - FU participants compared with baseline participants (1) | - Aims and hypotheses clearly derived from several different theoretical frameworks about child-related goal appraisals (1) | - validated measures used  (SR – NA; GB – no; WB – yes) (0.5)  -reliable measures used  (SR – NA; GB – no; WB – yes) (0.5) | - causal relationship between variables assessed (1)  - enough power to detect significant associations (1)  - all possible confounders assessed (0)  - statistics analysis plan (0) | 6  (average) |
| Thompson  (2011) | - sample included experiencing a blockage to parenthood goal (1)  - response rate (87% - 1)  - FU response rate (60% - 0)  - FU participants compared with baseline participants (1) | - Aims and hypotheses clearly derived from the theoretical assumptions of Adaptive Goal Adjustment (1) | - validated measures used  (SR - yes; GB – no; WB – yes) (0.66)  -reliable measures used  (SR –yes; GB – NA; WB – yes) (1) | - causal relationship between variables assessed (1)  - enough power to detect significant associations (0)  - all possible confounders assessed (1)  - statistics analysis plan (1) | 8.7  (average) |
| Kotter-Grühn (2009) | - sample included experiencing a blockage to parenthood goal (1)  - response rate (NM - 0)  - FU response rate (NA)  - FU participants compared with baseline participants (NA) | - Aims and hypotheses clearly derived from the theoretical assumptions of Adaptive Goal Adjustment (1) | -validated measures used  (SR – yes; GB – no; WB – yes) (0.66)  -reliable measures used  (SR –yes; GB – yes; WB – yes) (1) | - causal relationship between variables assessed (0)  - enough power to detect significant associations (1)  - all possible confounders assessed (1)  - statistics analysis plan (0) | 5.7  (average) |
| Light (2006) | - sample included experiencing a blockage to parenthood goal (0)  - response rate (NM - 0)  - FU response rate (NA)  - FU participants compared with baseline participants (NA) | - Aims and hypotheses clearly derived from the Action-phase Model of Developmental Regulation (1) | -validated measures used  (SR – no; GB – NA; WB – yes) (0.5)  -reliable measures used  (SR – yes; GB – NA; WB – yes) (1) | - causal relationship between variables assessed (0)  - enough power to detect significant associations (0)  - all possible confounders assessed (0)  - statistics analysis plan (1) | 3.5  (low) |
