# Supplementary material for: Self-Regulation and Wellbeing When Facing a Blocked Parenthood Goal: A Systematic Review and Meta-Analysis
Source: PLoS One. 2016 Jun 23;11(6):e0157649. doi: 10.1371/journal.pone.0157649 (PMC4919102; doi:10.1371/journal.pone.0157649)
Supplement: S4 Table — N, sample size; r, correlation coefficient; CI, Confidence Interval; LL, lower limit; UL, upper limit; p, significance level; NI, not investigated in the study; aGroup labels verbatim from studies; Urgent group, group of women approaching parenthood deadline; Passed group, group of women who missed parenthood deadline; ***p <. 001. (DOCX) [file pone.0157649.s006.docx]

|  |  | Blocked Goal with Wellbeing | | | | | | | | Blocked Goal with Self-Regulation Strategies | | | | | | |
| --- | --- | --- | --- | --- | --- | --- | --- | --- | --- | --- | --- | --- | --- | --- | --- | --- |
|  |  | Blocked Goal  with Negative Mood | | | | Blocked Goal  with Positive Mood | | | | Goal Disengagement | | | Goal Reengagement | | | |
| Studies | *N* | *r* | 95% CI [*LL, UL*] | | *p* | *r* | | 95% CI [*LL,UL*] | *p* | *r* | 95% CI [*LL, UL*] | *p* | *r* | 95% CI [*LL, UL*] | | *p* |
| Heckhausen  (study 1)  Urgent group^a^ | 51 | NI | | | | NI | | | | .42 | [.16, .62] | .002 | -.34 | [-.56, -.07] | | .01 |
| Heckhausen  (study 1)  Passed group^a^ | 43 | NI | | | | NI | | | | -.40 | [-.63, -.11] | .007 | -.45 | [-.66, -.17] | | .002 |
| Heckhausen  (study 2)  Urgent group^a^ | 47 | NI | | | | NI | | | | NI | | | NI | | | |
| Heckhausen  (study 2)  Passed group^a^ | 79 | NI | | | | NI | | | | NI | | | NI | | | |
| Kraaij (2009) | 83 | NI | | | | NI | | | | NI | | | NI | | | |
| Salmela-Aro (2008) | 97 | .35 | [.16, .51] | | <.001 | NI | | | | NI | | | NI | | | |
| Thompson (2011) | 47 | .29 | [.00, .53] | | .05 | -.10 | [-.38, .19] | | .51 | .05 | [-.24, .33] | .74 | - .29 | | [-.53, -.00] | .05 |
| Light (2006)  Urgent group^a^ | 29 | NI | | | | NI | | | | NI | | | NI | | | |
| Light (2006)  Passed group^a^ | 28 | NI | | | | NI | | | | NI | | | NI | | | |
| Kotter-Grühn (2009) | **168**  **102** | NI | | | | -.10 | [-.25, .05] | | .20 | .34 | [.16, .50] | <.001 | .09 | | [-.12, .28] | .37 |
| **Pooled estimate** |  | .33 | [.18, .47] | **<.001***** | | -.10 | [-.23, .04] | | .15 | .12 | [-.24, .44] | .53 | -.24 | | [-.48, .03] | **.**08 |
