# Supplementary material for: Self-Regulation and Wellbeing When Facing a Blocked Parenthood Goal: A Systematic Review and Meta-Analysis
Source: PLoS One. 2016 Jun 23;11(6):e0157649. doi: 10.1371/journal.pone.0157649 (PMC4919102; doi:10.1371/journal.pone.0157649)
Supplement: S5 Table — N, sample size; r, correlation coefficient; CI, Confidence Interval; LL, lower limit; UL, upper limit; p, significance level; NI, not investigated in the study; aGroup labels verbatim from studies; Urgent group, group of women approaching parenthood deadline; Passed group, group of women who missed parenthood deadline. (DOCX) [file pone.0157649.s007.docx]

|  |  | Goal Disengagement with Negative mood | | | Goal Reengagement with Negative mood | | | |
| --- | --- | --- | --- | --- | --- | --- | --- | --- |
| Studies | *N* | *r* | 95% CI  [*LL, UL*] | *p* | *r* | 95% CI  [*LL, UL*] | | *p* |
| Heckhausen (study 1)  Urgent group^a^ | 51 | .11 | [-.17, .37] | .44 | -.11 | [-.37, .17] | | .44 |
| Heckhausen (study 1)  Passed group^a^ | 43 | .38 | [.09, .61] | .01 | .14 | [-.17, .42] | | .37 |
| Heckhausen (study 2)  Urgent group^a^ | 47 | .32 | [.04, .56] | .03 | NI | | | |
| Heckhausen (study 2)  Passed group^a^ | 79 | -.26 | [-.46, -.04] | .02 | NI | | | |
| Kraaij (2009) | 83 | -.35 | [-.53, -.15] | .001 | -.01 | [-.23, .21] | | .93 |
| Salmela-Aro (2008) | 97 | NI | | | NI | | | |
| Thompson (2011) | 47 | -.21 | [-.47, .08] | .16 | -.44 | [-.65, -.18] | | .002 |
| Light (2006)  Urgent group^a^ | 29 | .23 | [-.15, .55] | .23 | NI | | | |
| Light (2006)  Passed group^a^ | 28 | .38 | [.01, .66] | .05 | NI | | | |
| Kotter-Grühn (2009) | 102 | NI | | | NI | | | |
| **Pooled estimate** |  | .06 | [-.17, .28] | .61 | -.11 | | [-.34, .13] | .37 |
