# Supplementary material for: Self-Regulation and Wellbeing When Facing a Blocked Parenthood Goal: A Systematic Review and Meta-Analysis
Source: PLoS One. 2016 Jun 23;11(6):e0157649. doi: 10.1371/journal.pone.0157649 (PMC4919102; doi:10.1371/journal.pone.0157649)
Supplement: S6 Table — N, sample size; r, correlation coefficient; CI, Confidence Interval; LL, lower limit; UL, upper limit; p, significance level; NI, not investigated in the study; aGroup labels verbatim from studies; Urgent group, group of women approaching parenthood deadline; Passed group, group of women who missed parenthood deadline;***p < .001. (DOCX) [file pone.0157649.s008.docx]

|  |  | Goal Disengagement with Positive Mood | | | | Goal Reengagement with Positive Mood | | | |  |
| --- | --- | --- | --- | --- | --- | --- | --- | --- | --- | --- |
| Studies | *N* | *r* | 95% CI  [*LL, UL*] | | *p* | *r* | 95% CI  [*LL, UL*] | *p* | | |
| Heckhausen (study 1)  Urgent group^a^ | 51 | .16 | [-.12, .42] | | .26 | .14 | [-.14, .40] | .33 | | |
| Heckhausen (study 1)  Passed group^a^ | 43 | -.09 | [-.38, .22] | | .57 | .37 | [.08, .60] | .01 | | |
| Heckhausen (study 2)  Urgent group^a^ | 47 | NI | | | | NI | | | |  |
| Heckhausen (study 2)  Passed group^a^ | 79 | NI | | | | NI | | | |  |
| Kraaij (2009) | 83 | .01 | | [-.21, .23] | .93 | .23 | [.02, .43] | .04 | | |
| Salmela-Aro (2008) | 97 | NI | | | | NI | | | | |
| Thompson (2011) | 47 | -.05 | | [-.33, .24] | .74 | .29 | [.00, .53] | .05 | | |
| Light (2006)  Urgent group^a^ | 29 | .01 | | [-.36, .38] | .96 | NI | | | | |
| Light (2006)  Passed group^a^ | 28 | .46 | | [.11, .71] | .01 | NI | | | | |
| Kotter-Grühn (2009) | 102 | .19 | | [-.01, .37] | .06 | .23 | [.04, .41] | | .02 | |
| **Pooled estimate** |  | .09 | | [-.03, .21] | .14 | .24 | [.14, .35] | | **<.001***** | |
