# Supplementary material for: Self-Regulation and Wellbeing When Facing a Blocked Parenthood Goal: A Systematic Review and Meta-Analysis
Source: PLoS One. 2016 Jun 23;11(6):e0157649. doi: 10.1371/journal.pone.0157649 (PMC4919102; doi:10.1371/journal.pone.0157649)
Supplement: S7 Table — k, number of studies; r, correlation coefficient; CI, confidence Interval; LL, lower limit; UL, upper limit; X2, chi-square; NA, not applicable because at least one of the groups only has one or no study; *p < .05. aCross-sectional and quasi-experimental studies were included in the same category in sensitivity analysis since both provide none or little evidence to infer causality. bThe quality of a study was categorized in the analyses as low, average or high according to the score obtained in the quality assessment. (DOCX) [file pone.0157649.s009.docx]

|  | Goal Disengagement with Negative Mood | | | | Goal Reengagement with Negative Mood | | | |
| --- | --- | --- | --- | --- | --- | --- | --- | --- |
|  | *k* | *r* | 95% CI  [*LL, UL*] | *X2* | *k* | *r* | 95% CI  [*LL, UL*] | *X2* |
| Nature of goal blockage  Type of blockage  Anticipated  Unanticipated  Degree of blockage    High  Low | 6  2 | .18  -.29 | [-.04, .38]  [-.58, .07] | **4.867*** | 2  2 | .01  -.22 | [-.36, .38]  [-.53, .15] | 0.753 |
|  | 4  4 | .01  .11 | [-.31, .33]  [-.22, .42] | 0.175 | 2  2 | .05  -.28 | [-.20, .30]  [-.51, -.02] | 3.194 |
| Study Design^a^  Cross-sectional or Quasi-experimental  Longitudinal | 7  1 | .10  -.21 | [-.16, .34]  [-.71, .43] | (NA) | 3  1 | -.00  -.44 | [-.15, .15]  [-.65, -.18] | (NA) |
| Study Quality^b^    Low  Average | 6  2 | .18  -.29 | [-.04 .38]  [-.58, .07] | **4.867*** | 2  2 | .01  -.22 | [-.36, .38]  [-.53, .15] | 0.753 |
