# Supplementary material for: Self-Regulation and Wellbeing When Facing a Blocked Parenthood Goal: A Systematic Review and Meta-Analysis
Source: PLoS One. 2016 Jun 23;11(6):e0157649. doi: 10.1371/journal.pone.0157649 (PMC4919102; doi:10.1371/journal.pone.0157649)
Supplement: S8 Table — k, number of studies; r, correlation coefficient; CI, Confidence Interval; LL, lower limit; UL, upper limit; X2, chi-square; NA, not applicable because at least one of the groups only has one or no study. aCross-sectional and quasi-experimental studies were included in the same category in sensitivity analysis since both provide none or little evidence to infer causality. bThe quality of a study was categorized in the analyses as low, average or high according to the score obtained in the quality assessment. (DOCX) [file pone.0157649.s010.docx]

|  | Goal Disengagement with Positive Mood | | | | Goal Reengagement with Positive Mood | | | |
| --- | --- | --- | --- | --- | --- | --- | --- | --- |
|  | *k* | *r* | 95% CI  [*LL, UL*] | *X2* | *k* | *r* | 95% CI  [*LL, UL*] | *X2* |
| Nature of goal blockage  Type of blockage  Anticipated  Unanticipated  Degree of blockage  High  Low | 4  2 | .13  -.02 | [-.07, .31]  [-.24, .22] | 0.820 | 2  2 | .25  .25 | [.04, .43]  [.08, .41] | 0.001 |
|  | 3  3 | .09  .05 | [-.14, .31]  [-.19, .28] | 0.079 | 2  2 | .28  .21 | [.11, .43]  [.01, .40] | 0.250 |
| Study Design^a^  Cross-sectional or quasi-experimental  Longitudinal | 6  1 | .12  -.05 | [-.02, .25]  [-.38, .29] | (NA) | 4  1 | .24  .29 | [.12, .35]  [.00, .53] | (NA) |
| Study Quality^b^  Low  Average | 4  3 | .13  .07 | [-.07, .32]  [-.12, .25] | 0.196 | 2  3 | .25  .24 | [.04, .43]  [.12, .36] | 0.002 |
